# Effects of resistance training combined with creatine supplementation on muscle strength, physical function, and muscle mass in older adults: a systematic review and three-level meta-analysis

**DOI:** 10.3389/fnut.2026.1919884

**Published:** 2026-08-25

**Authors:** Lingzi Yao, Doudou Yang, Jiajia Gao

**Affiliations:** 1 School of Physical Education, Guangzhou Maritime University, Guangzhou, China; 2 Guangdong University of Science and Technology, Dongguan, China; 3 Tianjin University of Sport, Tianjin, China

**Keywords:** creatine supplementation, resistance training, older adults, muscle strength, muscle mass, physical function, three-level meta-analysis

## Abstract

**Objective:**

To systematically evaluate the additional effects of creatine supplementation combined with resistance training, compared with resistance training alone, on muscle strength, muscle mass and physical function in older adults.

**Methods:**

PubMed, Embase, the Cochrane Library and Web of Science were searched from inception to 20 May 2026. Randomized controlled trials enrolling older adults aged 60 years and older and comparing creatine supplementation plus resistance training with resistance training alone or placebo plus resistance training were included. Three-level random-effects meta-analyses were conducted using the metafor package in R, allowing multiple correlated effect sizes from the same study to be retained while accounting for their statistical dependence. Risk of bias was assessed using the ROBUST-RCT tool, and the certainty of evidence was evaluated using the GRADE approach.

**Results:**

Eleven randomized controlled trials were included. Three-level meta-analysis showed that creatine supplementation combined with resistance training significantly improved muscle strength compared with resistance training alone, although the effect size was small (g = 0.31, 95% CI 0.18 to 0.45); the certainty of evidence for this outcome was moderate. The pooled effects on muscle mass (g = 0.35, 95% CI −0.09 to 0.78) and physical function (g = 0.47, 95% CI −0.08 to 1.03; *p* = 0.086) were not statistically significant, with low and very low certainty of evidence, respectively. Moderator analyses did not identify creatine dose, loading phase, intervention duration, training frequency, training intensity, sex or testing site as stable sources of effect variation. Sensitivity analyses indicated that the main findings were not materially influenced by any single effect size or study.

**Conclusion:**

In generally healthy older adults, creatine supplementation may serve as an adjunct to resistance training for modest improvements in muscle strength. Its additional effects on muscle mass and physical function remain uncertain. These findings should not be directly extrapolated to frail, sarcopenic or functionally limited older adults. Larger, well-reported randomized controlled trials are needed in these higher-risk older adult populations.

**Systematic review registration:**

https://www.crd.york.ac.uk/PROSPERO/view/, identifier CRD420261417231.

## Introduction

With the rapid aging of the global population, maintaining health in later life has become an important public health priority. According to the World Health Organization, the number of people aged 60 years and older exceeded the number of children younger than 5 years in 2020; by 2030, one in six people worldwide will be aged 60 years or older, with this population projected to increase from 1.0 billion in 2020 to 1.4 billion in 2030 and 2.1 billion by 2050 (1). Aging is commonly accompanied by declines in muscle strength, muscle mass and physical function, which can restrict activities of daily living and independent living, and increase the risk of disability, falls and other adverse health outcomes (2). Identifying safe and feasible strategies to maintain or improve muscle function in older adults is therefore essential for promoting healthy aging.

Among available interventions, resistance training is one of the best-supported non-pharmacological strategies for improving muscle function in older adults (3). The National Strength and Conditioning Association position statement indicates that appropriately designed resistance training can partly counteract age-related losses in muscle strength and muscle mass, while improving neuromuscular function, physical performance and activities of daily living (4). However, the adaptive response to resistance training in older adults is influenced by training intensity, training volume, intervention duration, nutritional status and individual health status, and the benefits of resistance training alone are not always consistent (5, 6). Accordingly, safe and feasible nutritional strategies used alongside resistance training may help further optimize improvements in muscle strength, muscle mass and physical function in this population (7).

Creatine supplementation is one of the most extensively studied sports nutrition strategies, and its potential effects are closely linked to the phosphocreatine system (8). The International Society of Sports Nutrition position stand indicates that creatine may increase intramuscular creatine and phosphocreatine stores, thereby supporting rapid ATP resynthesis during high-intensity muscle contractions and potentially improving training quality, training volume and recovery (9). This may be particularly relevant for older adults, in whom age-related declines in muscle function and alterations in energy metabolism may limit the response to training stimuli (10, 11). However, previous studies have shown inconsistent effects of creatine supplementation alone on muscle strength, muscle mass and physical function in older adults, and its benefits appear to be more evident when combined with resistance training (12, 13). Several randomized controlled trials have reported that creatine supplementation combined with resistance training provides additional benefits over resistance training alone for selected measures of muscle strength, fat-free mass, appendicular lean mass or functional performance (14–17). Nevertheless, previous reviews have also suggested that these effects may vary according to age, sex, dose, training protocol and baseline muscle function (18, 19).

Previous meta-analyses have suggested that creatine supplementation during resistance training may increase lean body mass and improve selected strength outcomes (20–22). However, many of these analyses classified participants with a mean age of 50 years or older as older adults, which limits the applicability of their findings to adults aged 60 years and older. In addition, prior analyses have mainly focused on lean body mass, chest press and leg press strength, with less attention to functional outcomes that more closely reflect daily activity capacity, such as chair stand performance, walking ability and timed up-and-go performance. Moreover, individual trials often report multiple correlated outcomes, and conventional meta-analytic approaches may not adequately account for dependency among effect sizes. Therefore, this study focused on adults aged 60 years and older and compared the effects of creatine supplementation combined with resistance training versus resistance training alone on muscle strength, muscle mass and physical function, using three-level meta-analysis to account for within-study dependency among effect sizes.

## Methods

This systematic review and meta-analysis was conducted in accordance with the methodological recommendations of the Cochrane Handbook for Systematic Reviews of Interventions and reported according to the PRISMA 2020 statement (23). The protocol was registered in PROSPERO (CRD420261417231).

### Data sources and search strategy

We systematically searched PubMed, Embase, the Cochrane Library and Web of Science to identify randomized controlled trials evaluating the effects of creatine supplementation combined with resistance training in older adults. Searches were conducted from database inception to 20 May 2026. Search terms were developed around creatine supplementation, resistance training, older adults and randomized controlled trials, including both controlled vocabulary and free-text terms such as “creatine,” “resistance training” and “older adults.” Boolean operators “OR” and “AND” were used to combine search terms. The full search strategies for each database are provided in Appendix 1.

In addition, the reference lists of relevant systematic reviews and meta-analyses were manually screened to identify potentially eligible studies not captured by the database searches. Records identified through reference checking were treated as records from other sources and underwent the same procedures for deduplication, title and abstract screening, and full-text eligibility assessment. Two reviewers independently screened titles and abstracts and assessed the full texts for eligibility. Any disagreements were resolved through discussion or, when necessary, consultation with a third reviewer.

### Eligibility criteria

Eligibility criteria were defined according to the Population, Intervention, Comparison, Outcome and Study design (PICOS) framework (24). Studies were included if they met the following criteria: (1) participants were aged ≥60 years; (2) the intervention group received creatine supplementation combined with resistance training; (3) the comparator group received placebo, non-creatine supplementation or another control treatment without creatine while completing the same resistance training program; (4) the study reported one or more eligible measures of muscle strength, physical function or muscle mass-related body composition. Muscle strength measures included upper- or lower-limb dynamic, isometric or isokinetic strength and handgrip; physical function measures included chair-rise performance, Timed Up and Go, gait-speed or walking tests, the 6-min walk test and floor-transfer tasks; and muscle mass-related outcomes included appendicular lean mass, skeletal muscle index, total lean or muscle mass, fat-free mass and lean tissue mass. All eligible measures reported within these domains were extracted and included in the primary analyses; (5) the study was a randomized controlled trial.

Studies were excluded if they met any of the following criteria: (1) they examined a single or acute dose of creatine supplementation, or assessed only acute exercise responses; (2) creatine was co-administered with other nutritional supplements, drugs or complex interventions and the independent effect of creatine could not be isolated; (3) the resistance training protocol was unclear, or the intervention consisted mainly of aerobic training, balance training, stretching or other non-resistance exercise; or (4) insufficient quantitative data were available and could not be obtained through figure extraction, statistical conversion or contact with the authors.

### Data extraction and coding

Data were extracted using a predesigned Microsoft Excel form. Extracted information included study characteristics, participant characteristics, intervention characteristics and outcome data. Study characteristics included first author, publication year, country and sample size. Participant characteristics included mean age, body mass index, sex and population type. Intervention characteristics included creatine supplementation protocol, use of a loading phase, supplementation dose, intervention duration, resistance training frequency, training intensity and resistance training program. Training intensity was standardized as percentage of one-repetition maximum (%1RM). When %1RM was directly reported, the original value was extracted; when training load was described using repetition maximum ranges, values were converted to %1RM according to the corresponding National Strength and Conditioning Association reference values and further coded as moderate or high intensity (25). Outcome data included measures of muscle strength, physical function and muscle mass.

For continuous outcomes, we preferentially extracted pre-to-post change scores and their standard deviations for the intervention and comparator groups. When change scores were not directly reported, mean change was calculated as: Mean change = Mean post − Mean baseline. When the standard deviation of the change score was not reported, it was estimated using the method recommended in the Cochrane Handbook (26):


$$SD_{\text{change}}=\sqrt{SD_{\mathrm{pre}}^{2}+SD_{\text{Post}}^{2}-2\times r\times SD_{\mathrm{pre}}\times SD_{\text{post}}}$$

where r denotes the correlation coefficient between baseline and post-intervention measurements. Because most included studies did not report this coefficient, we assumed *r* = 0.50, a commonly used value in meta-analyses of exercise interventions.

When the required means or standard deviations were not reported in the original articles, data were obtained, where possible, from figures, statistical conversions or Supplementary materials; if data remained unavailable, corresponding authors were contacted. For studies with multiple eligible intervention groups, comparator groups or outcome measures, data were extracted separately and effect sizes were calculated for each eligible comparison or outcome. To account for non-independence among multiple effect sizes from the same study, a three-level meta-analytic model was used, with effect sizes nested within studies.

### Risk of bias and certainty of evidence

Risk of bias in the included randomized controlled trials was assessed at the comparison and outcome levels using the ROBUST-RCT tool (27). For multi-arm trials, assessments were restricted to the comparison relevant to this review, namely creatine supplementation versus placebo or non-creatine control under the same resistance training program. The assessment covered six core domains: random sequence generation, allocation concealment, blinding of participants, blinding of intervention providers, blinding of outcome assessors and outcome data not included in the analysis. For each domain, methodological information reported in the study was first extracted and then judged for its potential to introduce bias. Overall risk of bias was determined using the worst-domain principle: a study was judged to be at low risk only when all core domains were rated as low risk, whereas any domain judged as possibly high risk or clearly high risk led to an overall high-risk rating.

The certainty of evidence for each outcome was assessed using the GRADE approach (24). Evidence from randomized trials was initially rated as high certainty and then downgraded according to risk of bias, inconsistency, indirectness, imprecision and publication or reporting bias. Upgrading was considered when the evidence met GRADE criteria for a large effect, dose–response gradient or plausible residual confounding that would reduce the observed effect. Certainty of evidence was classified as high, moderate, low or very low, reflecting the degree of confidence that the estimated effect approximates the true effect. Two reviewers independently completed the GRADE assessments for all outcomes. Disagreements were resolved through discussion or, when necessary, consultation with a third reviewer.

### Statistical analysis

Statistical analyses were performed using the metafor package in R. Continuous outcomes were expressed as standardized mean differences with small-sample correction, reported as Hedges’ g with 95% confidence intervals. The primary analysis used a three-level random-effects model, with effect sizes nested within studies, to account for dependency arising from multiple effect sizes contributed by the same study (28). Models were estimated using restricted maximum likelihood, with the random-effects structure specified as ~ 1 | study/effect size ID. The purpose of the three-level model was to partition variability into sampling error, within-study heterogeneity and between-study heterogeneity, thereby accounting for effect-size dependency rather than controlling for confounding (28).

Sensitivity analyses assessed the robustness of the three-level meta-analytic findings to assumptions regarding the baseline-to-post-intervention correlation, the handling of multiple effect sizes, outcome selection, risk of bias, and the influence of individual effect sizes and studies (Appendices 5–8). For effect sizes with r-dependent SD_change values, analyses were repeated using *r* = 0.25 and *r* = 0.75, with *r* = 0.50 retained for the primary analysis. We also aggregated effect sizes at the study level and fitted conventional two-level random-effects models using REML with Knapp–Hartung adjustment. To assess the influence of outcome selection, we retained one outcome measure per comparison dataset within each outcome domain and refitted conventional two-level random-effects models. As the retained measures were reported in common units across datasets, effects were expressed as mean differences: leg press performance for muscle strength, 30-s chair-stand performance for physical function, and lean mass for muscle mass. Cook’s distance, DFBETAS and hat values were used to assess the influence of individual effect sizes and studies (29). Finally, for muscle strength, we refitted the primary three-level model after excluding trials judged to be at overall high risk of bias.

Potential effect modifiers were explored using univariable three-level meta-regression or subgroup analyses, including creatine dose, loading phase, intervention duration, training frequency, training intensity, sex and testing site. Publication bias and small-study effects were assessed using funnel plots and Egger regression. Because individual studies could contribute multiple effect sizes, a three-level Egger regression was also conducted, decomposing the standard error into within-study and between-study components. For outcomes with an insufficient number of independent studies, publication bias analyses were interpreted descriptively only.

## Results

### Study selection

**Figure 1:**
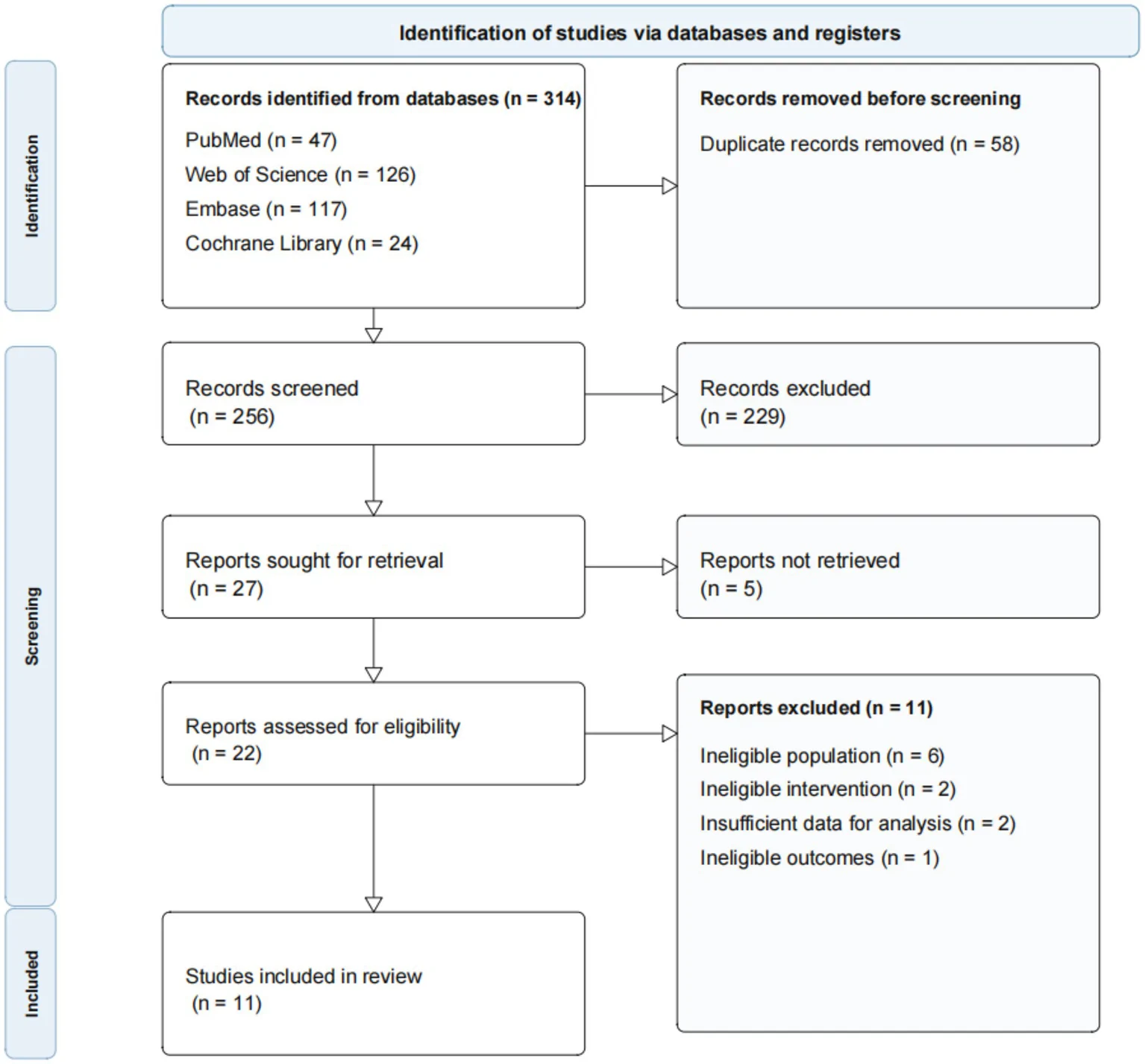
PRISMA flow diagram of study selection.

The database search identified 314 records. After removal of 58 duplicates, 256 records were screened by title and abstract, of which 229 were excluded. Twenty-seven full-text reports were sought for retrieval, and five could not be retrieved. The remaining 22 reports were assessed for eligibility. Eleven reports were excluded after full-text review, and 11 studies were ultimately included in this systematic review. The study selection process is shown in Figure 1.

### Characteristics of included studies

The included studies were published between 1998 and 2026 and were conducted in Canada (*n* = 4), Brazil (*n* = 4), Spain (*n* = 1), France (*n* = 1) and Iran (*n* = 1). All participants were adults aged 60 years or older, with a pooled mean age of 67.4 ± 6.5 years. Most studies enrolled healthy, sedentary or community-dwelling older adults; only one study included older adults with higher health risk or vulnerable status. Sex composition varied across the included comparisons: three comparisons included men only, three included women only and six included mixed-sex samples. Detailed characteristics of the included studies are presented in Table 1.

**Table 1:** Characteristics of the included studies.

Study	Group	N	Mean age, years	BMI, kg/m^2^	Country	Population	Sex	Creatine supplementation protocol	Resistance training intervention protocol
Bermon et al., 1998 (12)	RT + Cr	8	71.0 ± 5.4	24.7 ± 2.3	France	Healthy sedentary/moderately active older adults	Mixed	Creatine monohydrate was co-administered with glucose and dissolved in a warm or hot beverage: 20 g/day creatine plus 8 g/day glucose for 5 days (4 doses/day), then 3 g/day creatine plus 2 g/day glucose for 47 days.	8-week study; supervised RT for 7 weeks, 3 sessions/week on nonconsecutive days. Machine exercises focused on leg press, knee extension, and seated chest press. Training used 3 sets × 8 repetitions at approximately 80% 1RM with progressive high-intensity loading.
	RT + Pl	8	69.3 ± 1.1	25.0 ± 1.7				Glucose-only placebo, matched for flavor and appearance, dissolved in a warm or hot beverage: 7 g four times/day for 5 days, then 5 g/day for 47 days.	
Chrusch et al. (14)	RT + Cr	16	70.4 ± 6.4	27.9 ± 4.4	Canada	Sedentary/moderately active older men	Male	Creatine monohydrate (0.3 g/kg/day for 5 days, then 0.07 g/kg/day) was mixed with a sucrose-flour vehicle. Supplements were divided into 3 portions and consumed during or after meals.	12 weeks; 36 supervised sessions, 3 sessions/week. Whole-body RT with 12 exercises, 3 sets × 10 repetitions. Exercises included leg press, knee extension, bench press, lat pull-down, shoulder press, biceps curl, back extension, hip extension/flexion/adduction/abduction, and leg flexion. Initial intensity was ~50% pre-1RM for leg press, knee extension, and bench press and <50% pre-1RM for other exercises. Load was progressed by 5% or 2.2 kg when participants completed 10 reps on the third set; tapering was used before testing.
	RT + Pl	14	71.1 ± 6.7	25.7 ± 2.6				Sucrose-flour vehicle without creatine, with an equal portion of mixture replacing creatine; indistinguishable in flavor, texture, and appearance.	
Brose et al. (15)	RT + Cr	14	69.6 ± 5.3	NR	Canada	Healthy community-dwelling older adults	Mixed	Creatine monohydrate 5 g/day plus dextrose 2 g/day for 14 weeks; the supplement was dissolved in juice. Flavor and appearance were indistinguishable from placebo.	14 weeks; supervised RT 3 sessions/week on nonconsecutive days. Each session included 5-min warm-up and stretching. Twelve machine-based exercises trained major upper- and lower-body muscle groups in a circuit-set system. Participants performed 10 repetitions for arm exercises and 12 repetitions for the remaining exercises. Training progressed from 1 set at 50% initial 1RM to 3 sets at 80% 1RM. 1RM was re-evaluated every 2 weeks and loads were adjusted accordingly.
	RT + Pl	14	69.1 ± 4.5	NR				Dextrose 7 g/day for 14 weeks; the supplement was dissolved in juice and was indistinguishable in flavor and appearance.	
Chilibeck et al. (45)	RT + Cr	16	70.8 ± 6.6	NR	Canada	Sedentary/moderately active older men	Male	Creatine monohydrate 0.3 g/kg/day for 5 days, followed by 0.07 g/kg/day for 79 days, combined with a sucrose-flour vehicle.	12 weeks; supervised whole-body RT, 3 sessions/week. Participants performed 3 sets × 10 repetitions on 12 exercises: bench press, lat pull-down, shoulder press, leg press, biceps curl, back extensions, knee flexion, knee extension, and hip extension/flexion/abduction/adduction. Initial intensity was 50% of 1RM and loads were progressively increased once participants completed all 3 sets × 10 repetitions with proper form. Mean training attendance was about 95%.
	RT + Pl	13	71.5 ± 6.7	NR				Sucrose-flour vehicle without creatine, with an equal portion replacing creatine; indistinguishable in flavor, texture, and appearance.	
Candow et al. (46)	RT + Cr	13	65.5 ± 9.7	NR	Canada	Healthy older men	Male	Creatine monohydrate 0.1 g/kg on training days only for 10 weeks, provided in 3 equal doses. Each dose was mixed with chocolate- and cherry-flavored sucrose powder and dissolved in water immediately before training, immediately after training, and before bed.	10 weeks; supervised RT 3 sessions/week. Each session began with 5–10 min stationary cycling and light stretching. Participants performed 3 sets × 10 repetitions with 2-min rests. Initial intensity was ~70% 1RM for leg press and bench press and individual 10RM for other exercises. Exercises: bench press, leg press, lat pulldown, knee extension, shoulder press, leg curl, biceps curl, calf press, and triceps extension. Resistance was increased when all prescribed repetitions were completed with good form.
	RT + Pl	12	64.1 ± 10.7	NR				Chocolate- and cherry-flavored sucrose powder (1.2 g/kg on training days), divided into 3 doses and dissolved in water at the same times; matched for energy content, taste, texture, and appearance.	
Aguiar et al. (47)	RT + Cr	9	64 ± 4	NR	Brazil	Healthy older women	Female	Creatine monohydrate 5.0 g/day in capsules for 12 weeks. Both creatine and placebo capsules were consumed immediately after training with a carbohydrate drink.	12 weeks; supervised progressive RT, 3 sessions/week. Both groups performed the same whole-body RT program targeting major muscle groups. Outcomes were based on strength exercises including bench press, biceps curl, and knee extension; training volume increased over the program and functional tests included 30-s chair stand, arm curl, and get-up-from-floor tests.
	RT + Pl	9	65 ± 6	NR				Identical maltodextrin capsules, dose-matched to creatine, consumed immediately after training with the same carbohydrate drink.	
Alves et al. (48)	RT + Cr	12	66.4 ± 5.6	28.2 ± 1.7	Brazil	Healthy older women	Female	Creatine monohydrate 20 g/day for 5 days (4 × 5 g/day), then 5 g/day for the remaining 23 weeks. Participants were advised to consume supplements with meals.	24 weeks; supervised RT in an intrahospital gymnasium, 2 sessions/week, ~40 min/session. Training included 7 exercises: chest press, leg press, lat pull-down, leg extension, rowing, squat, and sit-ups. Protocol: 3 sets × 12–15RM, 1-min rest between sets. Load progression occurred when participants performed >15RM with proper technique in two consecutive sessions.
	RT + Pl	10	63.9 ± 3.8	26.5 ± 1.8				Dextrose at the same dosing schedule and with the same meal-based consumption advice.	
Gualano et al. (16)	RT + Cr	15	67.1 ± 5.6	28.0 ± 2.1	Brazil	Vulnerable postmenopausal women with osteopenia/osteoporosis	Female	Creatine monohydrate 20 g/day for 5 days (4 doses/day), then 5 g/day for 23 weeks. Supplements were preferably dissolved in juice; loading doses were taken with meals and at bedtime, and maintenance doses at lunch.	24 weeks; supervised RT, 2 sessions/week, monitored by fitness professionals in an intrahospital gymnasium. Exercises: leg press, leg extension, squat, seated row, bench press, lat-pull down, and sit-up. First week used reduced volume of 2 sets × 15–20RM; from week 2, training used 3 sets × 8–12RM. Absolute load was progressed when the participant could perform >12 repetitions on a given exercise set.
	RT + Pl	15	63.6 ± 3.6	28.2 ± 3.6				Dextrose at the same dosing schedule, preferably dissolved in juice and taken at the same times.	
Pinto et al. (17)	RT + Cr	13	67.4 ± 4.7	NR	Brazil	Healthy non-athletic older adults	Mixed	Creatine monohydrate 5 g/day for 12 weeks. On training days, it was consumed immediately after RT in a beverage containing 100 g lemon-flavored maltodextrin; on non-training days, it was consumed immediately after lunch.	12 weeks; supervised RT 3 sessions/week on Monday/Wednesday/Friday, between 3–4 p.m., ~60 min/session. Training was performed in a resistance-training laboratory, included mono- and multi-joint exercises, and progressed individually by increasing training volume (weight × sets × repetitions). A 1-week familiarization preceded training with 2 sets × 15RM for leg press, free squat, bench press, lat-pull down, abdominal floor crunch, and calf raise.
	RT + Pl	14	67.1 ± 6.3	NR				Maltodextrin 5 g/day, consumed using the same timing and, on training days, the same 100 g lemon-flavored maltodextrin beverage.	
Amiri & Sheikholeslami-Vatani (49)	RT + Cr	15	61.2 ± 7.06	27.84 ± 5.1	Iran	Healthy non-athletic older adults	Mixed	Creatine monohydrate 0.1 g/kg/day as a single dose, dissolved in a glass of water immediately after training; on non-training days, it was taken at 6:00 p.m.	10 weeks; RT 3 sessions/week with 1 day between sessions. Each session lasted ~70 min: 10-min warm-up, 50-min RT, 10-min cool-down. Exercises: leg extension, leg curl, barbell bench press, lateral pulldown, barbell curl, overhead press, and triceps extension machine. Protocol: 3 sets × 10 repetitions at 75% 1RM. Rest intervals were 2 min between sets and 3 min between exercises. 1RM was reassessed every 2 weeks to maintain intensity.
	RT + Pl	15	61.3 ± 5.7	28.37 ± 2.9				Maltodextrin placebo, consumed with the same timing and manner as the creatine supplement.	
Fernandez-Garrido et al., 2026a	RT + Cr	24	67.54 ± 2.99	26.71 ± 6.28	Spain	Sedentary community-dwelling older adults	Mixed	Creatine monohydrate 3 g/day plus maltodextrin 3 g/day for 16 weeks. The supplement was dissolved in water and taken each morning.	16 weeks; high-load, velocity-intentional elastic-band resistance training, 3 sessions/week (~60 min/session; 48 sessions). Sessions included 5–10 min warm-up, 40–50 min training, and 5–10 min cool-down. Protocol used 4 sets × 8 submaximal repetitions of multi-joint exercises, alternating upper and lower body; 2-min rest between sets and 90-s rest between exercises. Movement tempo emphasized maximal velocity-intent concentric action, 1-s isometric pause, and 2-s eccentric phase. Load/intensity was progressed using OMNI-RES RPE guidance.
	RT + Pl	21	69.10 ± 5.01	26.26 ± 5.43				Maltodextrin 6 g/day, matched to the total daily supplement weight, appearance, and taste; dissolved in water and taken each morning.	
Fernandez-Garrido et al. (50)	RT + Cr	13	70.05 ± 5.25	27.52 ± 6.66	Spain	Sedentary community-dwelling older adults	Mixed	Creatine monohydrate 3 g/day plus maltodextrin 3 g/day for 16 weeks. The supplement was dissolved in water and taken each morning.	16 weeks; aquatic high-load, velocity-intentional variable resistance training, 3 sessions/week (~60 min/session; 48 sessions). Sessions used the same basic structure as the land arm: 5–10 min warm-up, 40–50 min training, 5–10 min cool-down. Aquatic resistance training used high-velocity intent against water/hydrodynamic resistance; 4 sets × 8 submaximal repetitions, 2-min rest between sets and 90-s rest between exercises, with maximal velocity-intent concentric action, 1-s isometric pause, and 2-s eccentric emphasis where applicable. Intensity/progression followed OMNI-RES RPE guidance.
	RT + Pl	13	65.68 ± 3.44	25.78 ± 6.36				Maltodextrin 6 g/day, matched to the total daily supplement weight, appearance, and taste; dissolved in water and taken each morning.	

RT, resistance training; Cr, creatine; Pl, placebo.

### Risk of bias assessment

**Figure 2:**
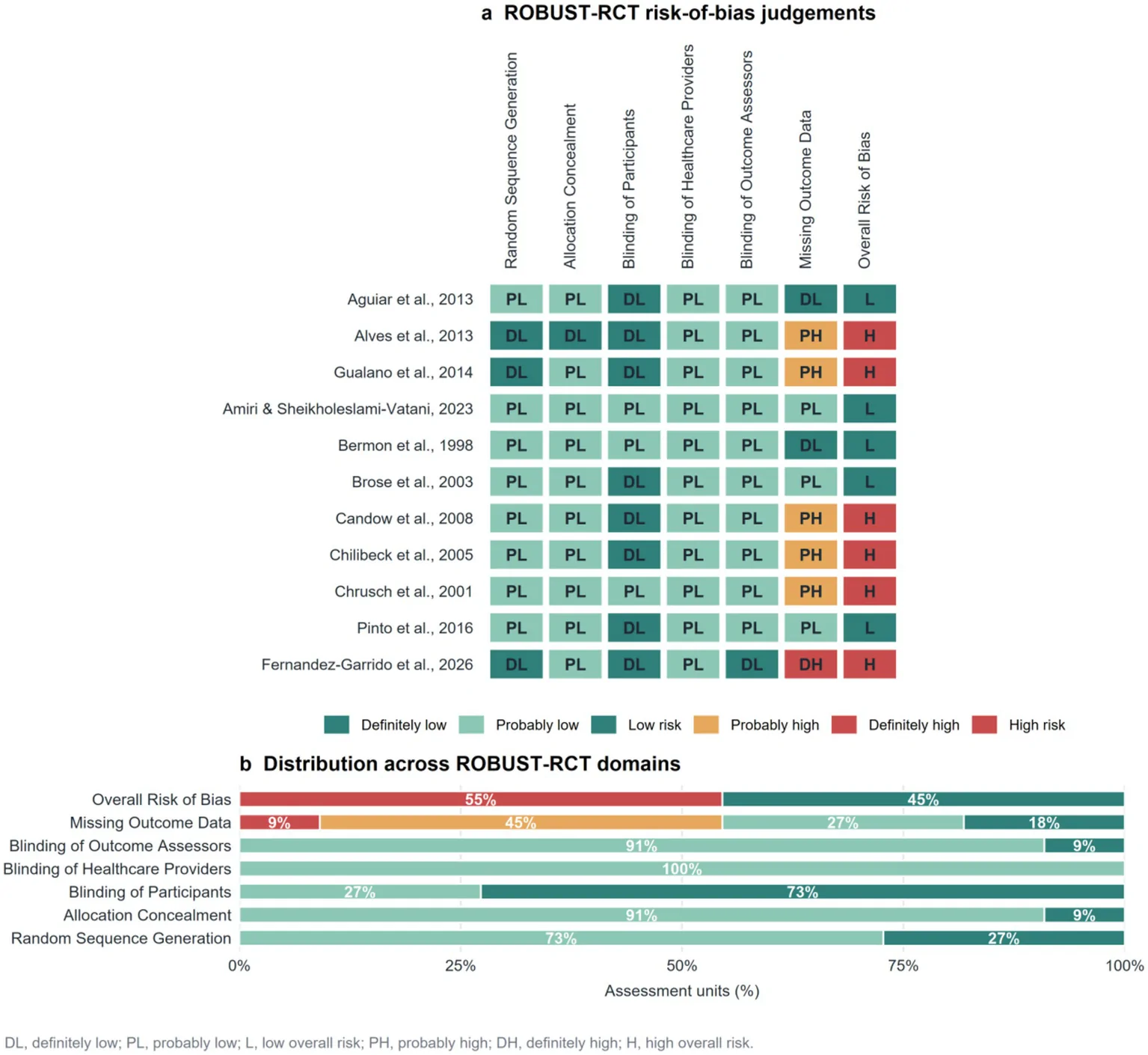
Risk-of-bias assessment of the included randomized controlled trials. **(a)** ROBUST-RCT risk-of-bias judgments across six core domains and overall risk of bias for the 11 included studies. **(b)** Distribution of risk-of-bias judgments across ROBUST-RCT domains.

Risk-of-bias assessment indicated generally acceptable methodological quality, although some studies were affected by missing outcome data (Figure 2). A total of 11 studies were assessed; five were judged to be at low risk of bias and six at high risk. Missing outcome data was the main source of high-risk judgments: one study was judged as definitely high risk and five as probably high risk in this domain. These judgments were mainly driven by high attrition, imbalance in missing data between groups, reduced sample size for specific outcomes, or withdrawals due to injury or illness that could have affected training completion and outcome measurement. In contrast, random sequence generation, allocation concealment, blinding of participants, blinding of intervention providers and blinding of outcome assessors did not lead to high-risk judgments. Three studies clearly reported the method used for random sequence generation, and one trial explicitly used central randomization. Although several studies did not fully report sequence generation or formal allocation concealment procedures, there was no evidence that group allocation could have been predicted or accessed before enrolment. Most studies used placebo-controlled double-blind supplementation protocols, and the included outcomes were mainly objective or performance-based; therefore, bias related to blinding was generally low. Detailed justifications for key judgments are provided in Appendix 2.

### Certainty of evidence

GRADE assessment showed moderate certainty of evidence for muscle strength, mainly downgraded because a substantial proportion of effect sizes came from studies at high risk of bias; no further downgrading was applied for inconsistency, imprecision or publication bias (Appendix 3). The certainty of evidence for physical function was very low, mainly because most effect sizes came from studies at high risk of bias, overall heterogeneity was statistically significant, and the confidence interval was wide and crossed the null line. The certainty of evidence for muscle mass was low, mainly because some included studies were at high risk of bias and the pooled confidence interval crossed the null line, indicating imprecision.

### Main outcomes

**Figure 3:**
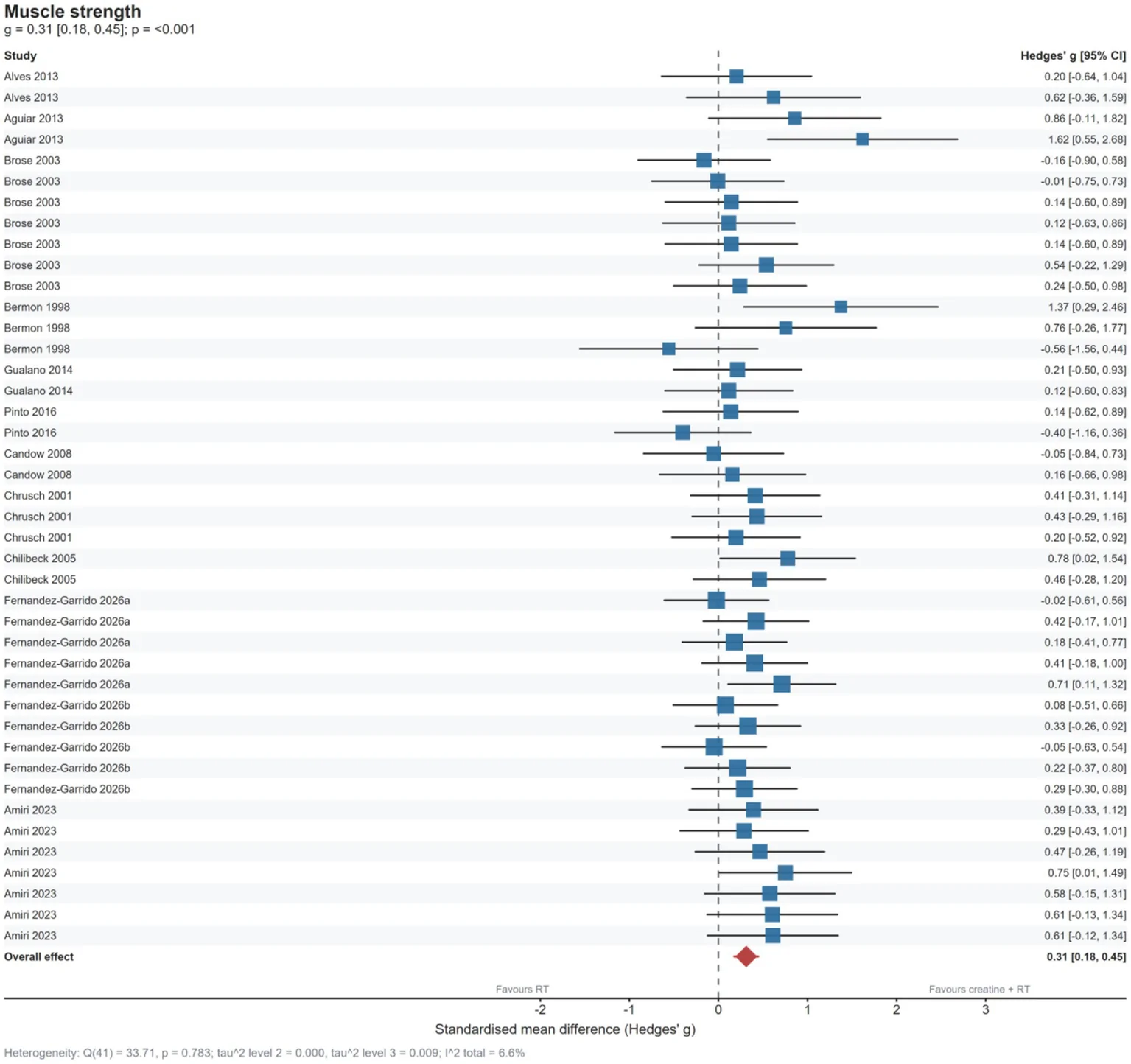
Forest plot for the effect on muscle strength.

For muscle strength, 42 effect sizes from 11 studies were included. The three-level random-effects model showed a significant positive effect of creatine supplementation combined with resistance training compared with resistance training alone (g = 0.31, 95% CI 0.18 to 0.45). Overall heterogeneity was not statistically significant, Q(41) = 33.71, *p* = 0.783. Variance component estimates showed no within-study variance at level 2 and a small between-study variance at level 3 (σ^2^ = 0.0095). Model comparisons showed that removing the level 2 variance component did not significantly reduce model fit (LRT = 0.00, *p* = 1.000), nor did removing the level 3 variance component (LRT = 0.31, *p* = 0.576). Thus, no substantial within-study or between-study heterogeneity was detected. The three-level structure was retained to account for dependency among multiple effect sizes contributed by the same study (Figure 3).

**Figure 4:**
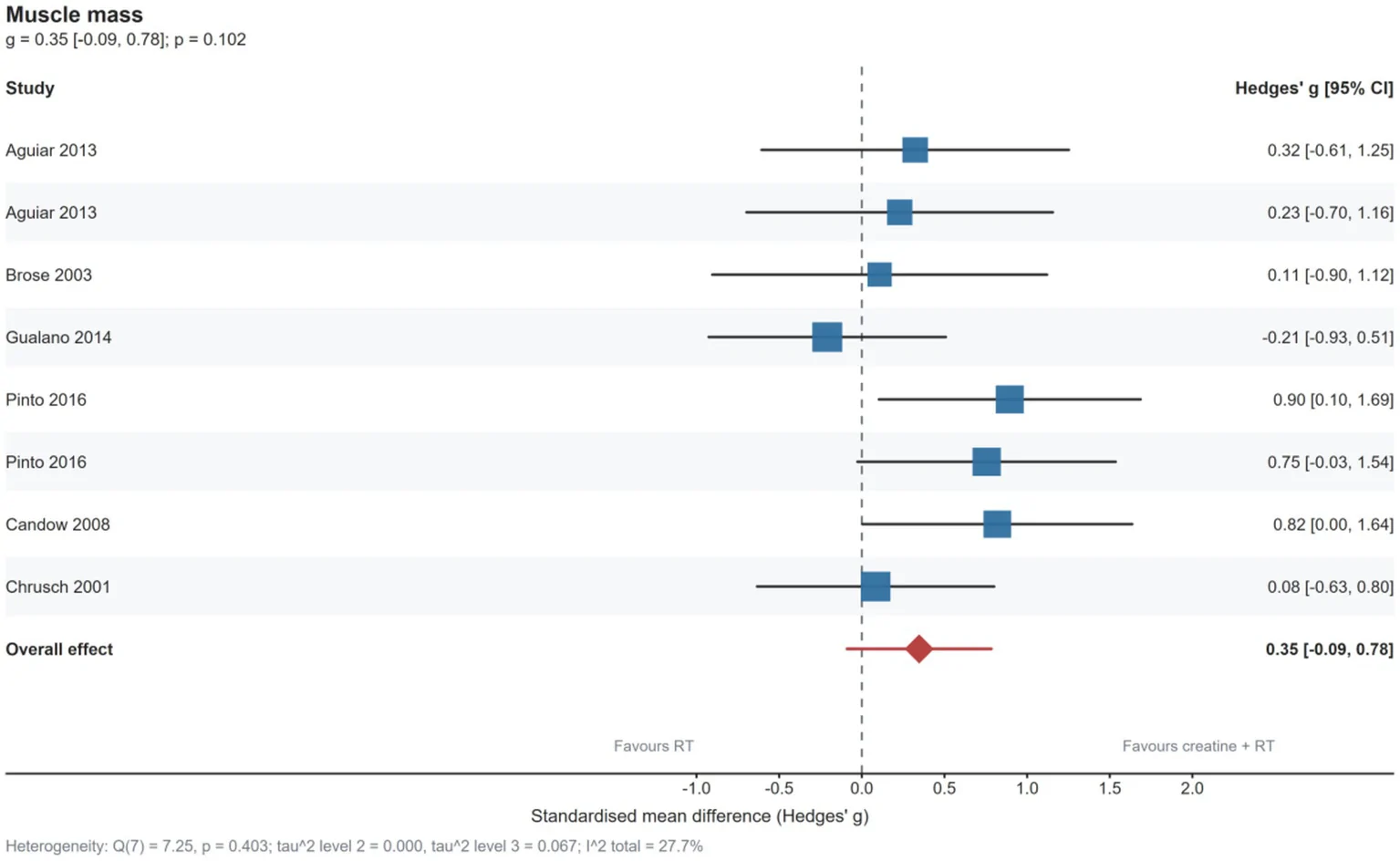
Forest plot for the effect on muscle mass.

For muscle mass, eight effect sizes from six studies were included. The pooled effect of creatine supplementation combined with resistance training was positive but not statistically significant (g = 0.35, 95% CI −0.09 to 0.78). Overall heterogeneity was not statistically significant, Q(7) = 7.25, *p* = 0.403. Variance component estimates showed no within-study variance at level 2 and a between-study variance of 0.0675 at level 3. Removing the level 2 variance component did not significantly reduce model fit (LRT = 0.00, *p* = 1.000), nor did removing the level 3 variance component (LRT = 0.53, *p* = 0.465), indicating no clear within-study or between-study heterogeneity (Figure 4).

**Figure 5:**
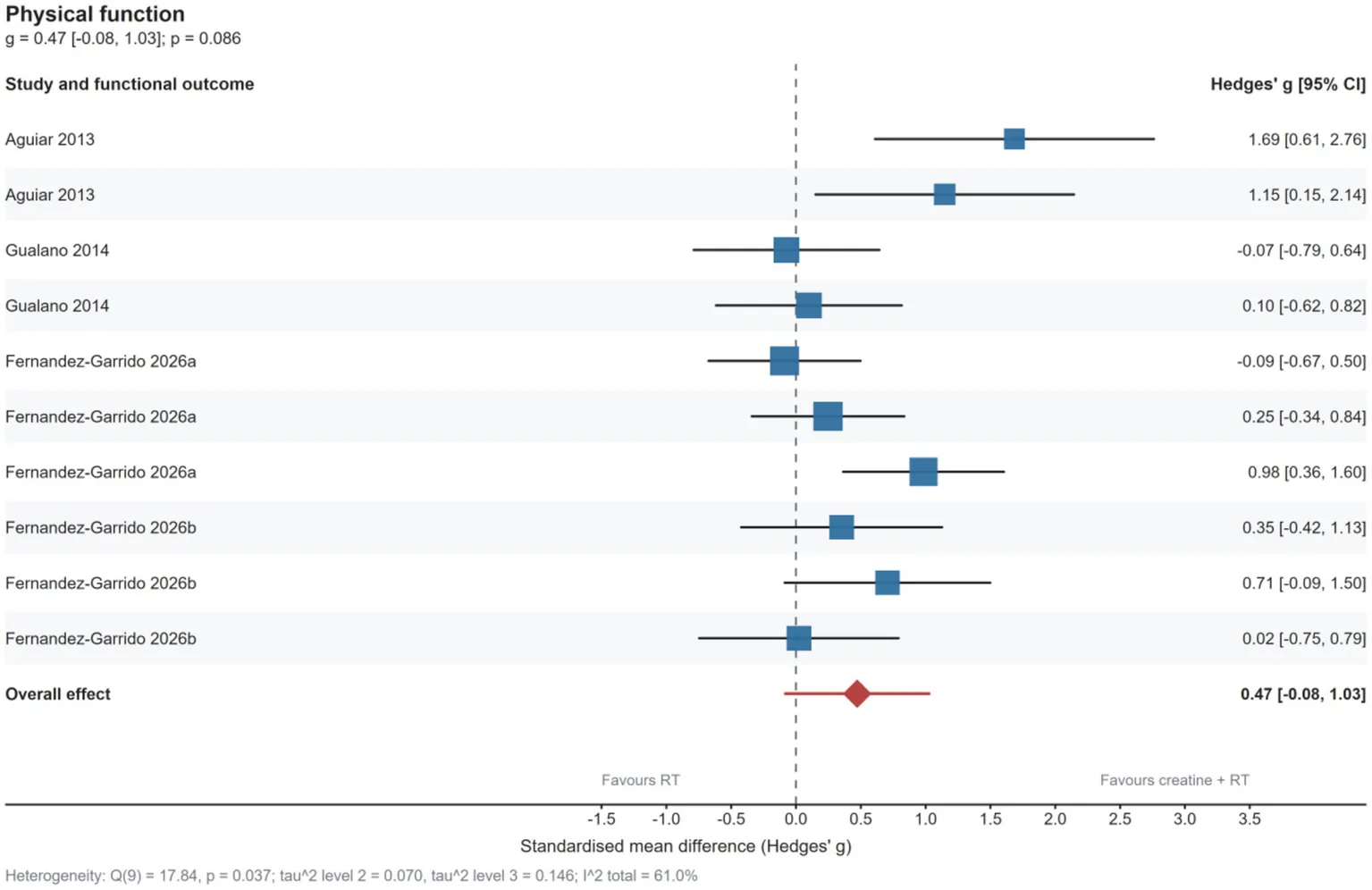
Forest plot for the effect on physical function.

For physical function, 10 effect sizes from three studies were included. The pooled effect was positive but not statistically significant (g = 0.47, 95% CI −0.08 to 1.03, *p* = 0.086). Overall heterogeneity was statistically significant, Q(9) = 17.84, *p* = 0.037. Variance component estimates showed a within-study variance of 0.0697 at level 2 and a between-study variance of 0.1459 at level 3. However, model comparisons showed that removing the level 2 variance component (LRT = 0.80, *p* = 0.371) or the level 3 variance component (LRT = 0.52, *p* = 0.471) did not significantly reduce model fit. These findings indicate statistically significant overall heterogeneity for physical function, although the available data were insufficient to clearly distinguish the independent contribution of within-study and between-study variance components (Figure 5).

### Moderator analyses

Univariable three-level meta-regression or subgroup analyses were conducted to explore potential modifiers of the intervention effect for muscle strength and muscle mass (Appendix 4). For muscle strength, loading phase, limb or testing site, creatine dose, intervention duration, training frequency and sex did not show significant moderating effects. The between-group difference by training intensity approached but did not reach statistical significance (*p* = 0.07), with a larger effect observed in the moderate-intensity group than in the high-intensity group; this finding should be interpreted cautiously. For muscle mass, creatine dose, training intensity, intervention duration and sex also showed no significant moderating effects, and residual heterogeneity was not statistically significant in any model. Overall, no clear or stable effect modifier was identified.

### Sensitivity analyses

Sensitivity analyses assessed the robustness of the three-level meta-analytic findings to assumptions regarding the baseline-to-post-intervention correlation, the handling of multiple effect sizes, outcome selection, risk of bias, and the influence of individual effect sizes and studies (Appendices 5–8). Varying the assumed correlation coefficient did not change the statistical interpretation of any outcome (Appendix 5, Table 5). Similarly, conclusions from conventional two-level random-effects meta-analyses following study-level aggregation were consistent with those from the primary three-level models (Appendix 6, Table 6). Outcome-specific analyses retaining one measure per comparison dataset within each domain led to the same statistical interpretation as the primary models (Appendix 8, Figures 17–19). Excluding the six trials judged to be at overall high risk of bias did not alter the conclusion for muscle strength (Appendix 7, Table 7).

Influence diagnostics were then conducted at both the effect-size and study levels. For muscle strength, one effect size from Aguiar 2013 was identified as a potential outlier (effectsizeID = 4; SMD = 1.619, 95% CI 0.554 to 2.683). However, its Cook’s distance, DFBETAS and hat value did not exceed the prespecified thresholds, and no highly influential study was identified. The pooled muscle-strength result was therefore not disproportionately influenced by any single effect size or study. For muscle mass, no potentially influential effect size was identified. At the study level, Chrusch 2001 exceeded the Cook’s distance threshold (0.642), with DFBETAS approaching the threshold (0.9999); however, its hat value did not exceed the threshold. Excluding this study yielded a positive but non-significant pooled effect (SMD = 0.397, 95% CI −0.125 to 0.919, *p* = 0.112), consistent with the primary analysis. For physical function, no potentially influential effect size was identified. Aguiar 2013 was potentially influential at the study level (Cook’s distance = 0.617; DFBETAS = 1.407), although its hat value did not exceed the threshold. After excluding this study, the pooled effect remained positive and non-significant (SMD = 0.280, 95% CI −0.064 to 0.623, *p* = 0.096). Overall, the sensitivity and influence analyses did not alter the direction or statistical interpretation of the pooled results.

### Publication bias

**Figure 6:**
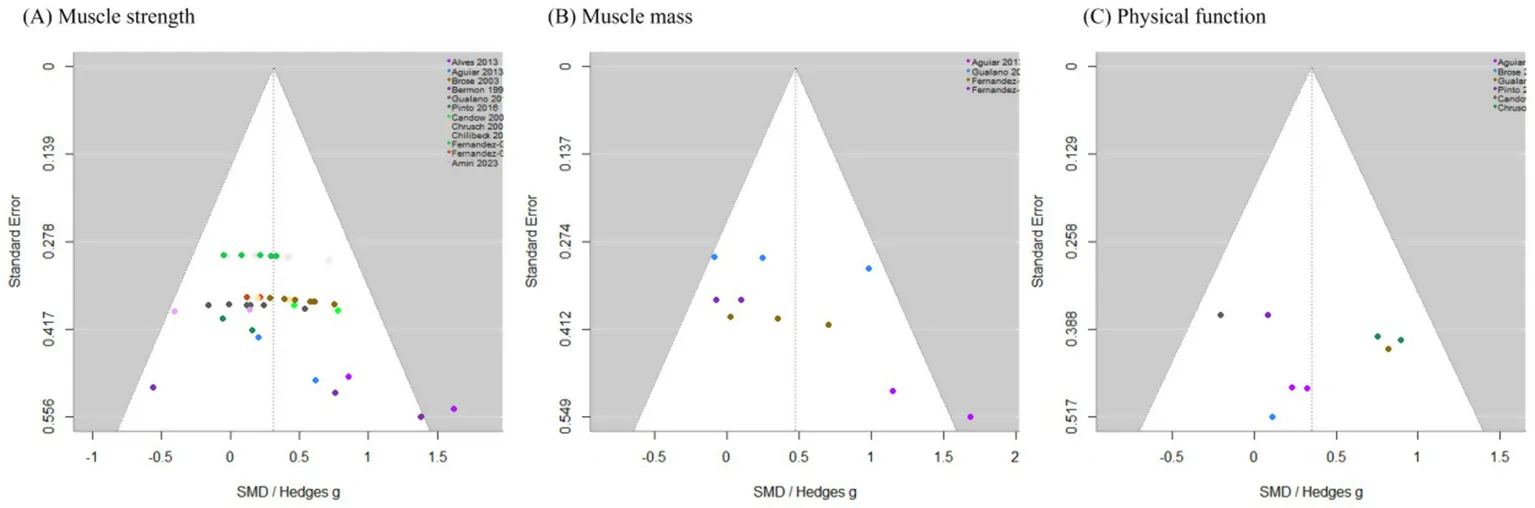
Funnel plot assessment of publication bias and small-study effects for **(A)** muscle strength, **(B)** muscle mass, and **(C)** physical function.

Potential publication bias was assessed using funnel plots and Egger regression (Figure 6). For muscle strength, small-study effects were assessed using three-level Egger regression. No significant small-study effect was detected (F (2, 39) = 0.9208, *p* = 0.4067), and neither the within-study nor between-study standard error component reached statistical significance, indicating no clear evidence of funnel plot asymmetry. For muscle mass and physical function, the number of independent studies was fewer than 10; therefore, Egger regression was not performed, and funnel plots were assessed descriptively. Visual inspection did not show obvious asymmetry, but given the limited number of studies, potential publication bias or small-study effects could not be ruled out.

## Discussion

This systematic review and meta-analysis included 11 randomized controlled trials comparing the effects of creatine supplementation combined with resistance training versus resistance training alone on muscle-related outcomes in adults aged 60 years and older. Based on moderate-certainty evidence, the three-level random-effects model showed that creatine supplementation combined with resistance training probably provides an additional improvement in muscle strength, although the effect size was small. No clear additional benefits were observed for muscle mass or physical function. The certainty of evidence was low for muscle mass and very low for physical function; therefore, these findings remain uncertain. Moderator analyses did not identify creatine dose, loading phase, intervention duration, training frequency, training intensity, sex or testing site as stable sources of effect variation. Overall, current evidence suggests that creatine supplementation may be an adjunct to resistance training for improving muscle strength in older adults. Its additional effects on muscle mass and physical function require confirmation in high-quality randomized trials.

The findings for muscle strength are generally consistent with previous evidence. Meta-analyses in middle-aged and older adults have broadly supported an additional effect of creatine supplementation during resistance training on muscle strength, although the magnitude of benefit has typically been small. Chilibeck et al. (21) reported small improvements in chest press strength (SMD = 0.35) and leg press strength (SMD = 0.24). Similarly, Liu et al. (22) found an improvement in lower-limb strength (SMD = 0.29), but not in upper-limb strength. This small but relatively consistent improvement in strength may arise first from changes in training performance rather than from marked increases in muscle mass. The creatine-phosphocreatine system helps maintain energy availability during repeated high-intensity contractions, which may allow individuals to complete prescribed loads, repetitions and training volume more effectively across multiple sets. Over several weeks of training, such cumulative differences in training quality may translate into greater strength adaptation.

Strength gains are generally smaller in older adults than in younger adults. In a meta-analysis of adults younger than 50 years, Wang et al. (30) reported larger absolute gains in upper- and lower-limb strength with creatine supplementation plus resistance training. These gains were larger than those reported in most studies involving adults older than 50 years (20, 31). This age-related difference may reflect the greater hypertrophic and neuromuscular adaptive potential of younger adults. In older adults, training responses may be constrained by age-related declines in muscle function, anabolic resistance and reduced recovery capacity (32, 33). Nevertheless, muscle strength is a clinically important target in later life, rather than simply a laboratory performance outcome. EWGSOP2 identifies low muscle strength as the key characteristic of probable sarcopenia and places strength assessment at the first step of case finding and diagnostic evaluation (34). In this context, the additional, albeit small, improvement in muscle strength observed with creatine supplementation may represent a clinically relevant adjunct benefit to resistance training in older adults.

No clear additional improvement in muscle mass was observed. Thus, the strength benefit associated with creatine plus resistance training was not necessarily accompanied by measurable increases in muscle mass. Strength changes may be influenced by neural drive, motor-unit recruitment, contraction efficiency and fatigue resistance, whereas increases in muscle mass usually require more sustained structural remodeling (33). In older adults, adaptation to resistance training may be expressed more through improvements in neuromuscular function than through short-term morphological change (35). Theoretically, creatine supplementation may support resistance-training-induced muscle remodeling through several mechanisms. These include increased cellular hydration, modulation of myogenic regulatory factors, effects on IGF-1/mTOR-related protein synthesis and reduced protein catabolism (36–38). However, in older adults, anabolic resistance, elevated inflammation, reduced recovery capacity and insufficient training stimulus may weaken the translation of these cellular responses into measurable increases in muscle mass. Previous meta-analyses have reported that creatine supplementation combined with resistance training can increase lean body mass compared with resistance training alone (21). However, lean body mass is not equivalent to skeletal muscle hypertrophy, and changes in this measure may be influenced by body water, glycogen storage and other non-fat tissue components. By contrast, Burke et al. (39) used MRI, CT and ultrasound to assess regional hypertrophy. They found only a small additional effect of creatine plus resistance training on muscle hypertrophy. Their age-moderation analysis also indicated slightly greater benefits in younger than in older participants, although the difference was limited. These findings suggest that positive results based on lean body mass may overestimate the effect of creatine on true muscle tissue accretion. Current evidence therefore more strongly supports creatine supplementation for improving resistance-training-related strength adaptation in older adults. Whether it can reliably increase muscle mass remains uncertain and should be tested using standardized, direct measurement methods.

This study also examined physical-function outcomes but found no clear additional benefit of creatine supplementation combined with resistance training over resistance training alone. The evidence was derived from only three trials contributing 10 effect sizes and encompassed clinically related but non-interchangeable tests, including chair-rise performance, Timed Up and Go, walking-based measures and floor-transfer tasks. These tests reflect different aspects of function, such as lower-limb functional performance, transitional mobility and balance, ambulatory capacity, and the ability to transfer from the floor. Thus, the observed heterogeneity may partly reflect variation in the functional constructs assessed rather than a single identifiable methodological or clinical source. The pooled estimate should therefore be interpreted as a broad summary across functional-performance measures, rather than as evidence of a uniform effect on every specific test.

Most included participants were healthy older adults, which may have limited the opportunity to detect functional gains. Gualano et al. (16) was one of the few trials involving postmenopausal older women; although the creatine plus resistance training group performed better than the placebo-only group in the 30-s chair-stand test, it did not differ significantly from the placebo plus resistance training group receiving the same training program. Changes in Timed Up and Go performance also did not differ between groups. These findings suggest that modest gains in muscle strength may not necessarily translate into detectable improvements in functional performance. According to the International Classification of Functioning, Disability and Health framework, functional status is shaped by interactions among health conditions, environmental factors and personal factors, rather than by muscle strength alone (40). Functional-performance tests also depend on lower-limb strength, balance, gait control, coordination, pain, comorbidities, cognitive status and habitual physical activity (41). Even if creatine supplementation produces small strength gains during resistance training, these gains may be insufficient to generate stable differences in chair-stand, Timed Up and Go, walking or floor-transfer performance (42). In addition, physical-function outcomes are sensitive to training specificity; when traditional resistance training does not adequately incorporate gait, balance, rapid sit-to-stand or task-oriented practice, transfer to these functional tasks may be limited (43). This issue may be particularly relevant in frail or sarcopenic older adults, in whom reduced muscle strength often coexists with limitations in balance, mobility and task-specific performance. Clinical practice guidelines for frail or sarcopenicl older adults recommend individually tailored multicomponent exercise programs that combine resistance, balance, aerobic and flexibility training (34, 44).

This study has several limitations. First, the number of included studies was limited, particularly for muscle mass and physical function outcomes. This restricted the precision of effect estimates, reduced the statistical power of moderator analyses, and limited the ability to evaluate publication bias and small-study effects. Second, moderator analyses assessed creatine dose, loading phase, intervention duration, training frequency, training intensity and sex. However, the number of available studies was limited. These analyses were underpowered and cannot exclude effect modification by these factors. Third, most included participants were generally healthy or relatively healthy older adults. Evidence specifically targeting frail, sarcopenic or functionally limited older adults remains scarce, and the findings should not be directly generalized to these higher-risk clinical populations. Fourth, delivery strategies varied across trials. Ten of the 11 trials administered creatine with a carbohydrate-containing vehicle or drink. Although comparators were generally matched, carbohydrate co-ingestion may influence creatine uptake. The limited and heterogeneous carrier categories precluded subgroup analysis; therefore, the pooled estimates may not fully generalize to creatine consumed without carbohydrate. Future studies should include larger, better-reported randomized controlled trials in adults aged 60 years and older. Particular attention should be given to frail, sarcopenic or functionally limited populations. Future trials should also clarify how creatine supplementation protocols, resistance-training characteristics and baseline health status influence intervention effects.

## Conclusion

This study suggests that, in generally healthy older adults aged 60 years and older, the additional benefit of creatine supplementation combined with resistance training is primarily observed for muscle strength, whereas no clear improvements were found for muscle mass or physical function. Creatine supplementation may therefore be considered an adjunct to support strength adaptation during resistance training, rather than an intervention assumed to increase muscle mass or improve physical function. These findings apply primarily to generally healthy older adults and should not be directly generalized to frail, sarcopenic or functionally limited older adults. Larger, rigorously designed randomized controlled trials are needed in these higher-risk older adult populations, particularly to clarify effects on muscle mass and physical function and the roles of supplementation protocols, resistance-training characteristics and baseline health status.

## Data Availability

The original contributions presented in the study are included in the article/Supplementary material, further inquiries can be directed to the corresponding author.
