# Supplementary material for: Effects of resistance training combined with creatine supplementation on muscle strength, physical function, and muscle mass in older adults: a systematic review and three-level meta-analysis
Source: Front Nutr. 2026 Aug 25;13:1919884. doi: 10.3389/fnut.2026.1919884 (PMC13549873; doi:10.3389/fnut.2026.1919884)
Supplement: Supplementary file 1 [file Supplementary_file_1.docx]

**Supplementary Appendix**

**Appendix 1: Search Strategies**

**Table 1.** Database-specific search strategy.

| **Database** | **Search string** |
| --- | --- |
| PubMed | ((((Creatine[MeSH Terms]) OR (creatine monohydrate[Title/Abstract])) OR (creatine supplement*[Title/Abstract])) OR (creatine loading[Title/Abstract])) OR (creatine ingestion[Title/Abstract]) |
|  | (((((((((((((((((((((((Training, Resistance[Title/Abstract]) OR (Strength Training[Title/Abstract])) OR (Training, Strength[Title/Abstract])) OR (Weight-Lifting Strengthening Program[Title/Abstract])) OR (Strengthening Programs, Weight-Lifting[Title/Abstract])) OR (Strengthening Program, Weight-Lifting[Title/Abstract])) OR (Weight Lifting Strengthening Program[Title/Abstract])) OR (Weight-Lifting Strengthening Programs[Title/Abstract])) OR (Weight-Lifting Exercise Program[Title/Abstract])) OR (Exercise Programs, Weight-Lifting[Title/Abstract])) OR (Exercise Program, Weight-Lifting[Title/Abstract])) OR (Weight Lifting Exercise Program[Title/Abstract])) OR (Weight-Lifting Exercise Programs[Title/Abstract])) OR (Weight-Bearing Strengthening Program[Title/Abstract])) OR (Strengthening Programs, Weight-Bearing[Title/Abstract])) OR (Strengthening Program, Weight-Bearing[Title/Abstract])) OR (Weight Bearing Strengthening Program[Title/Abstract])) OR (Weight-Bearing Strengthening Programs[Title/Abstract])) OR (Weight-Bearing Exercise Program[Title/Abstract])) OR (Exercise Programs, Weight-Bearing[Title/Abstract])) OR (Exercise Program, Weight-Bearing[Title/Abstract])) OR (Weight Bearing Exercise Program[Title/Abstract])) OR (Weight-Bearing Exercise Programs[Title/Abstract])) OR (Resistance training[MeSH Terms]) |
|  | ((((Aged[MeSH Terms]) OR (Elderly[Title/Abstract])) OR (old adult*[Title/Abstract])) OR (older women[Title/Abstract])) OR (older men[Title/Abstract]) |
| Web of Science | TS=("Creatine" OR "creatine monohydrate" OR "creatine supplementation" OR "creatine supplement" OR "creatine ingestion" OR "creatine loading") |
|  | TS=("Aged" OR "Elderly" OR "old adult" OR "old adults" OR "older women" OR "older men") |
|  | TS=("Resistance training" OR "Training, Resistance" OR "Strength Training" OR "Training, Strength" OR "Weight-Lifting Strengthening Program" OR "Strengthening Programs, Weight-Lifting" OR "Strengthening Program, Weight-Lifting" OR "Weight Lifting Strengthening Program" OR "Weight-Lifting Strengthening Programs" OR "Weight-Lifting Exercise Program" OR "Exercise Programs, Weight-Lifting" OR "Exercise Program, Weight-Lifting" OR "Weight Lifting Exercise Program" OR "Weight-Lifting Exercise Programs" OR "Weight-Bearing Strengthening Program" OR "Strengthening Programs, Weight-Bearing" OR "Strengthening Program, Weight-Bearing" OR "Weight Bearing Strengthening Program" OR "Weight-Bearing Strengthening Programs" OR "Weight-Bearing Exercise Program" OR "Exercise Programs, Weight-Bearing" OR "Exercise Program, Weight-Bearing" OR "Weight Bearing Exercise Program" OR "Weight-Bearing Exercise Programs") |
| Cochrane Library | MeSH descriptor: [Resistance Training] explode all trees |
|  | MeSH descriptor: [Creatine] explode all trees  ("creatine monohydrate":ti,ab,kw OR "creatine supplement":ti,ab,kw OR "creatine supplementation":ti,ab,kw OR "creatine ingestion":ti,ab,kw OR "creatine loading":ti,ab,kw) |
|  | MeSH descriptor: [Aged] explode all trees  ("Elderly":ti,ab,kw OR "old adult":ti,ab,kw OR "old adults":ti,ab,kw OR "older women":ti,ab,kw OR "older men":ti,ab,kw) |
| Embase | 'Resistance training'/exp OR 'Resistance training':ab,ti OR 'Training, Resistance':ab,ti OR 'Strength Training':ab,ti OR 'Training, Strength':ab,ti OR 'Weight-Lifting Strengthening Program':ab,ti OR 'Strengthening Programs, Weight-Lifting':ab,ti OR 'Strengthening Program, Weight-Lifting':ab,ti OR 'Weight Lifting Strengthening Program':ab,ti OR 'Weight-Lifting Strengthening Programs':ab,ti OR 'Weight-Lifting Exercise Program':ab,ti OR 'Exercise Programs, Weight-Lifting':ab,ti OR 'Exercise Program, Weight-Lifting':ab,ti OR 'Weight Lifting Exercise Program':ab,ti OR 'Weight-Lifting Exercise Programs':ab,ti OR 'Weight-Bearing Strengthening Program':ab,ti OR 'Strengthening Programs, Weight-Bearing':ab,ti OR 'Strengthening Program, Weight-Bearing':ab,ti OR 'Weight Bearing Strengthening Program':ab,ti OR 'Weight-Bearing Strengthening Programs':ab,ti OR 'Weight-Bearing Exercise Program':ab,ti OR 'Exercise Programs, Weight-Bearing':ab,ti OR 'Exercise Program, Weight-Bearing':ab,ti OR 'Weight Bearing Exercise Program':ab,ti OR 'Weight-Bearing Exercise Programs':ab,ti |
|  | 'Creatine'/exp OR 'creatine monohydrate':ab,ti OR 'creatine supplementation':ab,ti OR 'creatine supplement':ab,ti OR 'creatine ingestion':ab,ti OR 'creatine loading':ab,ti |
|  | 'Aged'/exp OR 'Elderly':ab,ti OR 'old adult':ab,ti OR 'old adults':ab,ti OR 'older women':ab,ti OR 'older men':ab,ti |

**Table 2.** Trial design, randomization unit, eligible comparisons, and dependency structure of the included randomized controlled trials.

| **Study** | **Trial design and randomization unit** | **Original arms** | **Eligible comparison(s) included in this review** | **Shared-control and dependency structure** |
| --- | --- | --- | --- | --- |
| Bermon et al., 1998 | 2×2 factorial parallel-group RCT; individual participant randomization; no crossover or cluster design reported | CON+Cr, CON+PL, RT+Cr, RT+PL | RT+Cr vs RT+PL | Non-training arms were excluded. No shared control was used in the retained comparison. |
| Chrusch et al., 2001 | Two-arm parallel-group RCT; individual participant randomization | Cr+RT, PL+RT | Cr+RT vs PL+RT | No shared control. Multiple strength and body-composition outcomes were extracted from the same comparison. |
| Brose et al., 2003 | Two-arm parallel-group RCT; individual participant randomization | Cr+RT, PL+RT | Cr+RT vs PL+RT | No shared control. Multiple strength outcomes were extracted from the same study. |
| Chilibeck et al., 2005 | Two-arm parallel-group RCT; individual participant randomization | Cr+RT, PL+RT | Cr+RT vs PL+RT | No shared control. Multiple outcomes were extracted from the same comparison. |
| Candow et al., 2008 | Three-arm parallel-group RCT; individual participant randomization | Cr+protein+RT, Cr+RT, PL+RT | Cr+RT vs PL+RT | The original placebo arm could theoretically serve as a common comparator for two creatine contrasts; however, only Cr+RT vs PL+RT was retained. The Cr+protein arm was excluded because the independent effect of creatine could not be isolated. |
| Aguiar et al., 2013 | Two-arm parallel-group RCT; individual participant randomization | Cr+RT, PL+RT | Cr+RT vs PL+RT | No shared control. Multiple strength, muscle-mass, and physical-function outcomes were extracted from the same comparison. |
| Alves et al., 2013 | Four-arm factorial parallel-group RCT; individual participant randomization | PL, Cr, PL+RT, Cr+RT | Cr+RT vs PL+RT | Non-training arms were excluded. No shared control was used in the retained comparison. |
| Gualano et al., 2014 | Four-arm factorial parallel-group RCT; individual participant randomization | PL, Cr, PL+RT, Cr+RT | Cr+RT vs PL+RT | Non-training arms were excluded. No shared control was used in the retained comparison. |
| Pinto et al., 2016 | Two-arm parallel-group RCT; individual participant randomization | Cr+RT, PL+RT | Cr+RT vs PL+RT | No shared control. Multiple muscle-mass and strength outcomes were extracted from the same comparison. |
| Amiri and Sheikholeslami-Vatani, 2023 | Three-arm parallel-group RCT; individual participant randomization | RT+Cr, RT+PL, non-training control | RT+Cr vs RT+PL | The non-training control group was excluded. No shared control was used in the retained comparison. |
| Fernandez-Garrido et al., 2026 | Six-arm parallel-group RCT; individual participant randomization | Elastic-band RT+Cr, elastic-band RT+PL, aquatic RT+Cr, aquatic RT+PL, control+Cr, control+PL | Elastic-band RT+Cr vs elastic-band RT+PL; aquatic RT+Cr vs aquatic RT+PL | The two non-training control arms were excluded. The two retained comparisons came from the same trial, not two independent studies, and were therefore treated as nested within the same study. |

**Appendix 2: Risk of Bias**

**Table 3.** ROBUST-RCT risk-of-bias judgments.

| **Author, year** | **Random sequence generation** | **Allocation concealment** | **Blinding of participants** | **Blinding of healthcare providers** | **Blinding of outcome assessors** | **Missing outcome data** | **Overall risk of bias** |
| --- | --- | --- | --- | --- | --- | --- | --- |
| Aguiar et al., 2013 | Probably low | Probably low | Definitely low | Probably low | Probably low | Definitely low | Low risk |
| Alves et al., 2013 | Definitely low | Definitely low | Definitely low | Probably low | Probably low | Probably high | High risk |
| Gualano et al., 2014 | Definitely low | Probably low | Definitely low | Probably low | Probably low | Probably high | High risk |
| Amiri & Sheikholeslami-Vatani, 2023 | Probably low | Probably low | Probably low | Probably low | Probably low | Probably low | Low risk |
| Bermon et al., 1998 | Probably low | Probably low | Probably low | Probably low | Probably low | Definitely low | Low risk |
| Brose et al., 2003 (Muscle strength) | Probably low | Probably low | Definitely low | Probably low | Probably low | Probably low | Low risk |
| Brose et al., 2003(Muscle mass) | Probably low | Probably low | Definitely low | Probably low | Probably low | Definitely high | High risk |
| Candow et al., 2008 | Probably low | Probably low | Definitely low | Probably low | Probably low | Probably high | High risk |
| Chilibeck et al., 2005 | Probably low | Probably low | Definitely low | Probably low | Probably low | Probably high | High risk |
| Chrusch et al., 2001 | Probably low | Probably low | Probably low | Probably low | Probably low | Probably high | High risk |
| Pinto et al., 2016 | Probably low | Probably low | Definitely low | Probably low | Probably low | Probably low | Low risk |
| Fernandez-Garrido et al., 2026 | Definitely low | Probably low | Definitely low | Probably low | Definitely low | Definitely high | High risk |

**Table 4.** Justifications for key ROBUST-RCT judgments.

| **Author, year** | **Overall risk of bias** | **Justification for key judgments** |
| --- | --- | --- |
| Aguiar et al., 2013 | Low risk | Participants were randomly assigned after baseline matching, but the sequence-generation method and a formal allocation-concealment procedure were not reported. Because baseline assessment preceded randomization and no open or predictable allocation list was reported, selection bias was judged probably low rather than high. Supplementation was masked with an identical placebo, the assessed outcomes were objective or performance-based, and all 18 randomized participants completed follow-up. |
| Alves et al., 2013 | High risk | The allocation sequence was computer-generated in blocks and implemented by central randomization. Supplement packages were coded until completion of the analyses, and the assessed comparison was restricted to creatine versus placebo within the same strength-training condition. Missing outcome data were the main concern: 6 of 28 randomized participants in the assessed comparison were not analyzed (PL+ST 4/14; CR+ST 2/14), making the overall risk of bias high. |
| Gualano et al., 2014 | High risk | The allocation sequence used a computer-generated block randomization code, whereas central allocation, SNOSE, sealed envelopes, or minimization safeguards were not reported. Supplement coding supports blinding after allocation, and the assessed comparison was creatine + RT versus placebo + RT with the same supervised training exposure and objective or performance-based outcomes. Attrition was balanced but substantial: 8 of 38 randomized participants in the assessed comparison were not analyzed (4/19 in each group), resulting in a high overall risk of bias. |
| Amiri & Sheikholeslami-Vatani, 2023 | Low risk | The report states that participants were randomly assigned in a double-blind design after eligible participants had been selected, although the sequence-generation and allocation-concealment procedures were not described. The placebo group consumed maltodextrin in the same manner as the creatine group, and the assessed outcomes were laboratory biomarkers and strength measures. Reported analyses used n=15 per group; no domain was judged at high risk of bias for the assessed comparison. |
| Bermon et al., 1998 | Low risk | Participants completed the initial strength assessment before random assignment, although a formal allocation-concealment method was not reported. Supplementation was conducted in a double-blind manner with very similar flavour and appearance, and the assessed outcomes were objective or performance-based. All 32 participants completed follow-up; no domain was judged at high risk of bias for the assessed comparison. |
| Brose et al., 2003 (Muscle strength) | Low risk | Participants were randomly assigned in a double-blind manner, although the sequence-generation method and allocation-concealment procedure were not reported. Supplement flavour and appearance were indistinguishable to participants and investigators, and the assessed outcomes were objective or performance-based. Two creatine-group participants withdrew for personal reasons unrelated to training or supplementation, leaving 28 participants for the main strength and biochemical analyses; no domain was judged at high risk of bias for these outcomes. |
| Brose et al., 2003 (Muscle mass) | High risk | Randomization and supplement blinding were the same as for the other Brose outcomes: assignment was described as random, but sequence generation and allocation concealment were not reported, and indistinguishable supplements were used in a double-blind design. The body-composition analysis was judged separately because the DXA-derived FFM/FM/%BF outcomes had substantially reduced denominators. Only 10 creatine and 6 placebo participants contributed these outcomes, so the substantial and imbalanced technical missingness drove the high overall risk of bias. |
| Candow et al., 2008 | High risk | Participants were randomized in a double-blind design, although the sequence-generation method and a formal allocation-concealment procedure were not reported. An individual not involved in the study prepared and labelled supplement bags, which supports blinding rather than direct evidence of allocation concealment; correct treatment identification was low, and the assessed outcomes were objective or performance-based. Missing outcome data were the main concern: 35 of 40 participants completed follow-up, with withdrawals concentrated in the creatine-plus-protein and placebo groups and smaller denominators for some urinary biomarkers. |
| Chilibeck et al., 2005 | High risk | Participants provided written informed consent before random assignment, although the sequence-generation and allocation-concealment procedures were not described. The placebo mixture was indistinguishable from the creatine mixture in flavour, texture, and appearance, and DXA-derived bone outcomes were objective. The overall high-risk judgment was driven by missing outcome data: three participants withdrew and one placebo participant was excluded after randomization, leaving 16 creatine and 13 placebo participants for analysis. |
| Chrusch et al., 2001 | High risk | Participants were randomly assigned during the baseline period after providing written informed consent, although sequence generation and allocation concealment were not described. The assessed outcomes were objective or performance-based, and both groups followed the same RT program. The overall high-risk judgment was driven by missing outcome data because 3 of 33 assigned participants withdrew due to recurrent knee or back injuries that prevented adherence to training and testing. |
| Pinto et al., 2016 | Low risk | Participants were randomized to creatine + RT or placebo + RT, although the sequence-generation method and a formal allocation-concealment procedure were not reported. Supplement packages were coded so that investigators and participants were unaware of contents until completion of the intervention, and the assessed outcomes were objective or performance-based. Five of 32 randomized participants withdrew for personal reasons, with similar losses between groups (CR+RT 3/16; PL+RT 2/16); this was judged unlikely to materially bias the effect estimate. |
| Fernandez-Garrido et al., 2026 | High risk | An independent researcher generated a computer-based randomization sequence and assigned alphanumeric codes, although a central allocation system, SNOSE, sealed-envelope procedure, or minimization safeguard was not described. Participants were blinded to supplementation, outcome evaluators and data analysts were blinded, and the assessed outcomes were biomarkers, strength/function tests, and cognitive test performance. The main limitation was attrition: 150 participants were randomized, 112 started the intervention, and 103 completed; baseline-observation-carried-forward imputation does not remove the high risk arising from large and imbalanced missingness. |

**Appendix 3: Certainty of Evidence**

**Table 5.** GRADE summary of findings for the main outcomes.

| **Outcome** | **No. of studies (effect sizes)** | **Study design** | **Risk of bias** | **Inconsistency** | **Indirectness** | **Imprecision** | **Other considerations** | **No. of participants RT+Cr** | **No. of participants RT** | **Absolute effect, 95% CI** | **Certainty** | **Importance** |
| --- | --- | --- | --- | --- | --- | --- | --- | --- | --- | --- | --- | --- |
| Muscle strength | 11 (42) | Randomized trials | Serious¹ | Not serious | Not serious | Not serious | None | 179 | 166 | SMD 0.31 higher, 95% CI 0.18 to 0.45 higher; small improvement | Moderate | Critical |
| Physical function | 3 (10) | Randomized trials | Serious² | Serious³ | Not serious | Serious⁴ | None | 61 | 58 | SMD 0.47 higher, 95% CI 0.08 lower to 1.03 higher; effect may range from no effect to large improvement | Very low | Critical |
| Muscle mass | 6 (8) | Randomized trials | Serious⁵ | Not serious | Not serious | Serious⁶ | None | 76 | 70 | SMD 0.35 higher, 95% CI 0.09 lower to 0.78 higher; possible small improvement | Low | Important |

**Explanations**

1. **Risk of bias, muscle strength:** Downgraded one level because 19 of 42 effect sizes were derived from studies rated as high risk of bias, indicating that study limitations contributed substantially to the evidence base. We did not downgrade by two levels because effect estimates from low-risk and high-risk studies were directionally consistent, and sensitivity analyses did not identify any single effect size or study that materially changed the pooled result.
2. **Inconsistency, physical function:** Downgraded one level because statistically significant heterogeneity was detected in the overall analysis (Q = 17.84, p = 0.037). Variance component estimates suggested variability at both the within-study and between-study levels (level 2 σ² = 0.0697; level 3 σ² = 0.1459), although likelihood ratio tests did not clearly support the independent contribution of either variance component. Given the small number of included studies and the absence of a clear explanation for the observed heterogeneity, the certainty of evidence was downgraded for inconsistency.
3. **Inconsistency, physical function:** Downgraded one level because statistically significant heterogeneity was detected in the overall analysis (Q = 17.84, p = 0.037), and residual heterogeneity remained significant after subgroup analysis by creatine dose (QE = 17.67, p = 0.024). Although likelihood ratio tests did not clearly attribute heterogeneity to either within-study or between-study variance components, the small evidence base limited the reliability of these variance component tests.
4. **Imprecision, physical function:** Downgraded one level because the 95% CI crossed the line of no effect and was wide (SMD 0.47, 95% CI -0.08 to 1.03), including both little or no effect and a potentially large beneficial effect. The evidence was also based on only 119 participants from 4 studies, further reducing confidence in the precision of the estimate.
5. **Risk of bias, muscle mass:** Downgraded one level because several included studies were rated as high risk of bias in a small evidence base. Although the direction of effect generally favored RT+Cr, the limited number of studies meant that high-risk studies could still meaningfully affect the certainty of the pooled estimate.
6. **Imprecision, muscle mass:** Downgraded one level because the 95% CI crossed the line of no effect (SMD 0.35, 95% CI -0.09 to 0.78), indicating that the true effect may range from little or no effect to a moderate or large improvement. The sensitivity analysis excluding Chrusch 2001 remained statistically non-significant, supporting continued uncertainty around the estimate.

**Appendix 4: Influence Diagnostics**

**Figure 1.** Study-level influence diagnostics for the muscle strength meta-analysis.

**Figure 2.** Effect-size-level influence diagnostics for the muscle strength meta-analysis.

**Figure 3.** Study-level influence diagnostics for the muscle mass meta-analysis.

**Figure 4.** Effect-size-level influence diagnostics for the muscle mass meta-analysis.

**Figure 5.** Effect-size-level influence diagnostics for the physical function meta-analysis.

**Figure 6.** Study-level influence diagnostics for the physical function meta-analysis.

Appendix 5: Sensitivity Analyses for the Assumed Pre–Post Correlation

Only SD_change values estimated from baseline and post-intervention standard deviations were recalculated. All models retained the same three-level random-effects structure, effect-size definition and restricted maximum likelihood estimator.

Table 6. Sensitivity of pooled estimates to the assumed pre–post correlation coefficient.

| **Outcome** | **r = 0.25** | **r = 0.50 (primary)** | **r = 0.75** | **Statistical interpretation** |
| --- | --- | --- | --- | --- |
| **Muscle strength** | g = 0.277 95% CI 0.157 to 0.397 P < 0.001 | g = 0.31 95% CI 0.18 to 0.45  P < 0.001 | g = 0.399 95% CI 0.238 to 0.560 P < 0.001 | Significant in all analyses |
| **Muscle mass** | g = 0.340 95% CI −0.094 to 0.773 P = 0.106 | g = 0.35 95% CI −0.09 to 0.78  P = 0.106 | g = 0.363 95% CI −0.085 to 0.812 P = 0.097 | Non-significant in all analyses |
| **Physical function** | g = 0.369 95% CI −0.033 to 0.771 P = 0.068 | g = 0.47 95% CI −0.08 to 1.03 P = 0.086 | g = 0.680 95% CI −0.159 to 1.519 P = 0.1 | Non-significant in all analyses; estimate and heterogeneity varied |

Forest plots for the correlation-coefficient sensitivity analyses are presented below.

**Assumed correlation coefficient: r = 0.25**

Figure 7. Correlation-coefficient sensitivity analysis for muscle strength (r = 0.25).

Figure 8. Correlation-coefficient sensitivity analysis for muscle mass (r = 0.25).

Figure 9. Correlation-coefficient sensitivity analysis for physical function (r = 0.25).

**Assumed correlation coefficient: r = 0.75**

Figure 10. Correlation-coefficient sensitivity analysis for muscle strength (r = 0.75).

Figure 11. Correlation-coefficient sensitivity analysis for muscle mass (r = 0.75).

Figure 12. Correlation-coefficient sensitivity analysis for physical function (r = 0.75).

Appendix 6: Alternative Models for Dependent Effect Sizes

Effect sizes within studies or independent comparison datasets were aggregated using inverse-variance weighting under a compound-symmetry working correlation of rho = 0.60. Conventional two-level random-effects models were then fitted by restricted maximum likelihood with Knapp–Hartung adjustment.

Table 7. Study-level aggregation followed by conventional two-level random-effects meta-analysis.

| **Outcome** | **No. of aggregated units** | **Hedges’ g (95% CI)** | **Statistical interpretation** |
| --- | --- | --- | --- |
| **Muscle strength** | 12 | 0.30 (0.13 to 0.47) | Statistically significant |
| **Muscle mass** | 6 | 0.32 (−0.14 to 0.78) | Not statistically significant |
| **Physical function** | 4 | 0.41 (−0.40 to 1.22) | Not statistically significant |

Figure 13. Study-level aggregated two-level random-effects model for muscle strength.

Figure 14. Study-level aggregated two-level random-effects model for muscle mass.

Figure 15. Study-level aggregated two-level random-effects model for physical function.

Appendix 7: Sensitivity Analysis Excluding Trials at Overall High Risk of Bias

The primary three-level model for muscle strength was refitted after excluding Alves 2013, Gualano 2014, Candow 2008, Chilibeck 2005, Chrusch 2001 and Fernandez-Garrido 2026, which were judged to be at overall high risk of bias.

Table 8. Muscle-strength sensitivity analysis excluding trials at overall high risk of bias.

| **Outcome** | **Effect sizes / studies** | **Hedges’ g (95% CI)** | **P value** | **I²** |
| --- | --- | --- | --- | --- |
| **Muscle strength** | 21 / 5 | 0.394 (0.008 to 0.780) | 0.046 | 41.4% |

Figure 16. Three-level meta-analysis of muscle strength after exclusion of trials at overall high risk of bias.

Appendix 8: Outcome-Specific Sensitivity Analyses

To assess whether the broad outcome domains were sensitive to the selection of individual measures, the primary three-level models were repeated after retaining one outcome measure per comparison dataset within each domain. These analyses were expressed as mean differences in the original measurement units for leg press performance, 30-s chair-stand performance and lean mass.

Figure 17. Three-level meta-analysis retaining leg press performance for muscle strength (mean difference).

Figure 18. Three-level meta-analysis retaining 30-s chair-stand performance for physical function (mean difference).

Figure 19. Three-level meta-analysis retaining lean mass for muscle mass (mean difference).
